# Exosomal 15-LO2 mediates hypoxia-induced pulmonary artery hypertension in vivo and in vitro

**DOI:** 10.1038/s41419-018-1073-0

**Published:** 2018-10-03

**Authors:** Min Zhang, Wei Xin, Cui Ma, Hongyue Zhang, Min Mao, Ying Liu, Xiaodong Zheng, Lixin Zhang, Xiufeng Yu, Huajian Li, Daling Zhu

**Affiliations:** 1 College of Pharmacy, Harbin Medical University, Harbin, 150081 P. R. China; 2 Central Laboratory of Harbin Medical University (Daqing), Daqing, 163319 P. R. China; 3 College of Medical Laboratory Science and Technology, Harbin Medical University (Daqing), Daqing, 163319 P. R. China; 4 Department of Pathophysiology, Harbin Medical University (Daqing), Daqing, 163319 P. R. China

## Abstract

Our previous studies have shown that 15-LO2/15-HETE induced by hypoxia played an important role in pulmonary arterial hypertension (PH). However, the transportations of 15-LO2/15-HETE among the cells remain elusive. In this study, we investigated the specific involvement of 15-LO2-containing exosomes in the overproliferation of pulmonary artery endothelial cells (PAECs) induced by hypoxia and the underlying mechanism. In vitro, 15-LO2 was abundantly expressed and enriched in exosomes secreted from hypoxic PAECs, which subsequently activated the STAT3 signaling pathway, resulting in a robust increase in PAECs proliferation. In vivo treatment with the exosomes inhibitor GW4869 protected the pulmonary vascular homeostasis from dysfunctional and abnormal remodeling. Moreover, 15-LO2 was ubiquitinated under hypoxia, and further inhibition of the ubiquitin-proteasome system significantly suppressed PAECs proliferation, suggesting that ubiquitination of 15-LO2 may contribute to its sorting into exosomes. Overall, these findings indicate a previously unrecognized effect of exosomes and the cargo 15-LO2 in pulmonary vascular homeostasis on the pathogenesis of PH.

## Introduction

Pulmonary artery hypertension (PH) is a life-threatening disease characterized by the pathological manifestation of pulmonary vascular remodeling, which leads to enhanced right ventricular afterload, right heart failure, and eventually death^1^. Increased cell proliferation and survival in the intima of vascular wall are major cellular events resulting in pulmonary vascular remodeling^2,3^. However, the underlying mechanisms involved in the dysfunction of pulmonary artery endothelial cells (PAECs) in pulmonary vascular homeostasis and the progression of PH remain unclear.

Exosomes are membrane-bound extracellular vesicles, 30–150 nm in diameter, which are generated via an endocytic pathway^4^. And contain a range of functional biomolecules (lipids, proteins, mRNAs, and miRNAs), perform functions locally within a defined microenvironment (paracrine or autocrine action) or enter the circulation to act at distal sites (endocrine action)^5–7^. It has been reported that exosomes secret from cancer stem cells communicated with normal cells in an endocrine manner to suppress the immune response and have profound effects on metastasis^8–10^. Evidence also shows that exosomes from mesenchymal stromal cells in a paracrine manner inhibit inflammation in PH^11^. However, it is unclear whether exosomes serve as extracellular vesicles in a paracrine or autocrine manner to participate in the dysfunction of PAECs in pulmonary vascular homeostasis, as well as the cellular origin of exosomes and the role of PAEC-derived exosomes in the process of PH.

The lipid-peroxidizing enzyme 15-lipoxygenase2 (15-LO2), which preferentially metabolizes arachidonic acid to 15-hydroxyeicosatetraenoic acid (15-HETE), is widely distributed in the pulmonary vasculature^12^. Our previous studies have suggested that 15-LO2 was abundantly expressed in PAECs and pulmonary artery smooth muscle cells under hypoxia, and the upregulation of 15-LO2 subsequently exerted an effect on cell proliferation, anti-apoptosis activities, and vasoconstrictive physiological milieu, ultimately leading to PH^13–16^. Given the distribution of 15-LO2 in the cytoplasm of PAECs under hypoxia, we propose that the interplay between 15-LO2 and exosomes in the cell cytoplasm may modulate pulmonary artery remodeling in PH.

Here, we report that hypoxia increases the secretion of exosomes that exert their pro-proliferative function on recipient PAECs in a paracrine or autocrine manner, which is attributed at least partially to the enrichment of 15-LO2 in exosomes and then activate the STAT3 signaling pathway. Our data shed new light on the cargoes of exosomes and their subsequent influence on PH.

## Materials and methods

### Animals and lung tissues preparation

Animal care and use conformed to the Guide for the Care and Use of Laboratory Animals and was conducted in compliance with the NIH guidelines. 15-LO knockout mice were purchased from Jackson Laboratory (Stock No:002778, USA), and adult male C57BL/6 mice (25 g) were obtained from the Experimental Animal Center of Harbin Medical University. The mice were randomized to 21 days of normal and hypoxic environments with fractional inspired oxygen (FiO_2_) 0.21 and 0.12, respectively. Normoxic mice were kept in the same room adjacent to the hypoxic chamber. To test the effect of GW4869 on hypoxia, mice were intraperitoneally administered with GW4869 (1.25 mg/kg) or DMSO, as control vehicle, once a day for 21 days under normoxic and hypoxic environments. At the end of the 21 days, the lung tissues were harvested for morphological examinations.

### Histological and morphometric analyses

The lung tissues of mice were fixed in 4% paraformaldehyde for 24 h, and then dehydrated, cleared, and embedded in paraffin wax. The paraffin-embedded tissues were sliced into 5-μm-thick sections and stained with hematoxylin and eosin (HE) or Masson’s trichrome stain. For immunohistochemistry, the lung tissues were dewaxed and incubated overnight with α-smooth muscle actin (α-SMA) at a dilution of 1:100. Afterward, the tissues were washed three times with PBS and then incubated with secondary antibodies. Following incubation, the tissue sections were stained with 3, 3-diaminobenzidine (DAB) and restrained with hematoxylin. Images of tissues were captured with a fluorescence microscope (Nikon) equipped with a digital camera.

### Measurement of right ventricular systolic pressure (RVSP) and ventricular weight

RVSP was measured by right heart catheterization. The catheter was inserted into the right ventricle (RV), and RVSP was continuously recorded for 30 min. For ventricular weight measurement, mice hearts were excised and RV free wall was dissected and weighed. The degree of RV hypertrophy was determined as the ratio of RV weight to left ventricular (LV) weight plus that of the septum (RV/LV+S).

### Microfil perfusion

The lung tissues were rinsed with normal saline, and then fixed with formalin for several minutes. The vasculature of the fixed lung tissues was infused with microfil (MV-122; Flow Tech, Inc., Massachusetts, USA). Alcohol methyl salicylate clearing was implemented according to the manufacturer’s instructions.

### Echocardiography

Mice were anesthetized with 4% chloral hydrate. Echocardiography was performed with a Vevo2100 imaging system (Visual Sonics Inc., Toronto, ON, Canada) and a 30 MHz probe. The pulmonary arterial velocity time integral (PAVTI), pulmonary arterial pre-ejection time, and pulmonary arterial ejection time were obtained from stable images.

### Cell isolation and culture

The study was approved by the Ethical Committee of Laboratory Animals at Harbin Medical University. PAECs were collected from calf lungs, which were obtained from a local slaughterhouse. The detailed method used was previously described^17^. PAECs were cultured in 20% fetal bovine serum (FBS)-DMEM in a 5% CO_2_ humidified incubator at 37 °C.

### Small interfering RNA (siRNA) design and transfection

PAECs were transfected with siRNA, which was designed and synthesized by GenePharma (China). Non-targeted control siRNA was used as a negative control (NC). The siRNA sequences were as follows: 15-LO2 (NM153301.2): 5′-GCAAUGAAGAACGCCAAAUTT-3′ and NC: 5′-CCUACGCCACCAAUUUCGU-3′. The transfection methods were used previously described^15^.

### Measurement of 15-HETE level

Exosomes were purified from a culture solution of PAECs, and the amount of endogenous 15-HETE was measured with 15(S)-HETE enzyme immunoassay (EIA) Kit (Catalog No. 534721, Cayman). The results were analyzed with Cayman Chemical Company EIA Tools.

### Exosomes purification, characterization

For the isolation and quantification of released exosomes, PAECs were cultured to 90% confluence in a complete medium. Afterwards, the PAECs were washed twice with PBS and then incubated in DMEM with 5% exosome-depleted FBS for 24 h. The culture medium was harvested for exosomes isolation according to the manufacturer’s protocol of ExoQuickTC Exosome Precipitation kit. Exosomes were verified by electron microscopy and western blot analysis. The final exosomes pellet was resuspended in PBS. Exosomes were labeled with PKH26 (Sigma Aldrich) according to the manufacturer’s protocol. After PKH26 staining, the exosomes were collected by ExoQuickTC Exosome Precipitation kit (SBI). Finally PKH26-labeled exosomes were resuspended in PBS.

### Transmission electron microscopy (TEM), immuno-electron microscope (IEM), and NanoSight analysis (NTA)

For TEM and IEM, the prepared exosomes were resuspended in PBS. Counterstaining and photographing of exosomes were performed by the Electron Microscopy Center of Harbin Veterinary Research Institute. For NTA, exosomes extracted from PAECs were dissolved in PBS, and the measurement was completed by Tai Chang HuaJia commercial co. LTD (China).

### Tube formation assay

PAECs in 96-well plates (Costar, Corning) were covered with growth factor-reduced Matrigel (BD Biosciences) in a total volume of 30 μl, and then cultured. Next, the required reagents were added to the medium of different wells. Tube formation was photographed under a fluorescence microscope (Nikon).

### MTT assay

PAECs were cultured in 96-well plates and then treated with the required reagents in DMEM with 5% FBS. Then, the samples were exposed to hypoxia (3% O_2_) for 24 h. At the end of the incubation at 37 °C, the PAECs were incubated for another 4 h in a medium containing 0.5% 3-[4, 5-dimethylthiazol-2-yl]-2, 5-diphenyl-tetrazolium bromide (MTT). The reaction was terminated by the addition of DMSO to the medium. The absorbance at 540 nm was measured using a spectrophotometer.

### Migration assay

For the modified Boyden chamber migration assay, PAECs were cultured in the upper chamber of a transwell, which was inserted into 24-well plates. The lower chamber was filled with 20% FBS–DMEM. Migration was measured after incubation in 0.4% crystal violet in 10% ethanol. The number of migrated PAECs was measured by counting the number of stained nuclei per high-power field under a microscope (Nikon).

### BrdU incorporation assay

After pretreatment with the indicated agents in DMEM supplemented with 5% FBS for 24 h, cultured PAECs in 96-well culture plates were incubated with 5-BrdU labeling solution for ∼2 h, followed by FixDenat for 30 min. Then, removed the FixDenat solution and added to anti-BrdU-POD solution for 90 min. The antibody conjugate was removed by rinsing with wash solution, and the PAECs were placed in substrate solution.

### Immunofluorescnce staining

PAECs were cultured on glass coverslips. At 24 h after exposure with the indicated agents, the PAECs were fixed with 4% paraformaldehyde, permeabilized with 0.01% Triton X-100, blocked with 3% normal bovine serum, and then incubated with the following appropriate antibodies at 4 °C overnight: CD31 (1:100; Santa Cruz Biotechnology), Ki67 (1:100; Abcam), CD63 (1:100; Abcam), p-STAT3 (1:100; Abcam), and HRS (1:100; China). After washing with PBS, the PAECs were incubated with the appropriate FITC-conjugated secondary antibody, Cy3-conjugated secondary antibody, and DAPI in the dark. Images were captured by confocal laser scanning microscopy.

### Co-immunoprecipitation

The samples were lysed in a lysis buffer (Tris 50 mM, pH 7.4, NaCl 150 mM, Triton X-100 1%, EDTA 1 mM, and PMSF 2 mM) and then incubated with monoclonal antibody (5 μg). Afterward, the cells lysates were added with Protein A/G beads (Santa Cruz Biotechnology) overnight at 4 °C. Antibody–protein complexes were washed three times with PBS, then the buffer was removed, and the pellet was resuspended in protein loading buffer (2×). The eluted samples were then subjected to western blot analysis.

### Statistical analysis

Data are presented as means ± SD. Statistical analysis between different groups was performed with Student’s *t* test or one-way ANOVA. *p* < 0.05 was considered statistically significant.

## Results

### Hypoxia upregulates exosomes secretion

**Fig. 1:**
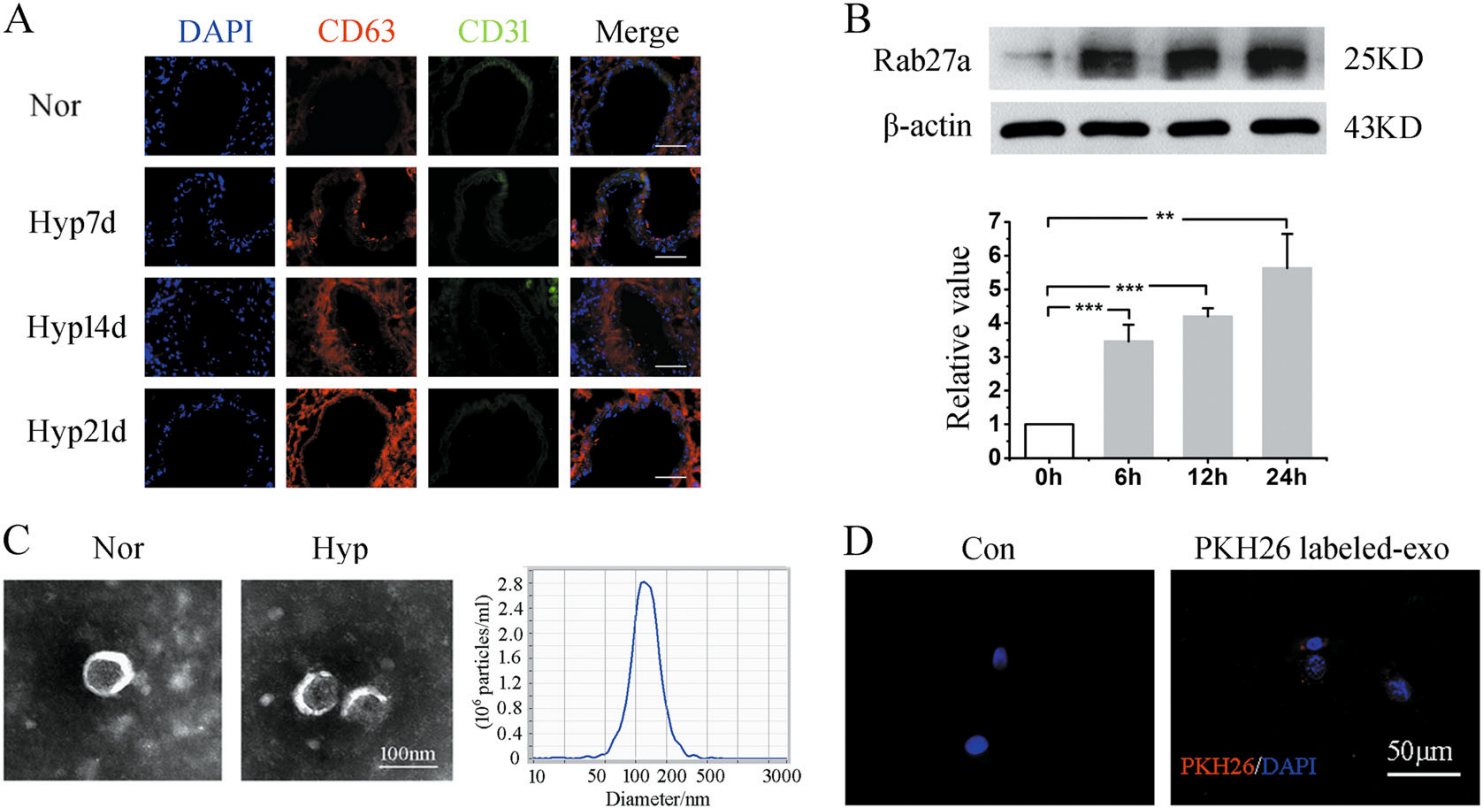
Exosomes were highly secreted under hypoxia. **a** Immunofluorescence staining analysis was done to detect the cellular expression of CD63 in the endothelial layer of lung tissues from relative mice. Scale bar = 100 μm. **b** Western blot of Rab27a administrated with hypoxia. **c** Exosomes extracted from PAECs were prepared for electron microscopy assay. The image showed cup-shape morphology of exosomes, and the particle size of the vesicles was measured by NanoSight analysis. Scale bar = 100 nm. **d** Exosomes from PAECs were labeled with PKH26 and then added to PAEC cultures. Scale bar = 50 μm. Con, control; Nor, normoxia; Hyp, hypoxia; Hyp7d, hypoxia 7 days; Hyp14d, hypoxia 14 days; Hyp21d, hypoxia 21 days. **p *<* 0.01 and ***p *<* 0.001. All values are presented as mean ± SD

Firstly, the effect of hypoxia on exosomes secretion was detected. As shown in Fig. 1a, CD63, a protein biomarker of exosomes, was highly expressed in a time-dependent manner in the intima of pulmonary arteries of hypoxic mice, as compared with the normal group. Immunofluorescnce staining demonstrated that the exosomes were widely distributed in the intima of the pulmonary arteries. To test whether hypoxia affected the secretion of exosomes in vitro, PAECs were exposed to hypoxia. We found that hypoxia induced a time-dependent increase of the Rab27a (a regulated protein of exosomes secretion) expression with peaks at 24 h after hypoxia exposure (Fig. 1b). TEM revealed the “cup-shaped” vesicles of exosomes with a median diameter of ~140 nm, and there was an increase in exosomes quantity in the PAECs under hypoxia (Fig. 1c). These results partly indicate that hypoxia promoted exosomes secretion. Next, we tested whether these PAEC-derived exosomes can be taken up by PAECs. They were labeled with the red dye PKH26 and then added into the culture medium of PAECs. At last, the presence of red fluorescence staining in these cells, which indicated the PAECs exhibited efficient uptake of the PAEC-derived exosomes (Fig. 1d).

### Exosomes are involved in the progression of hypoxia-induced PH

**Fig. 2:**
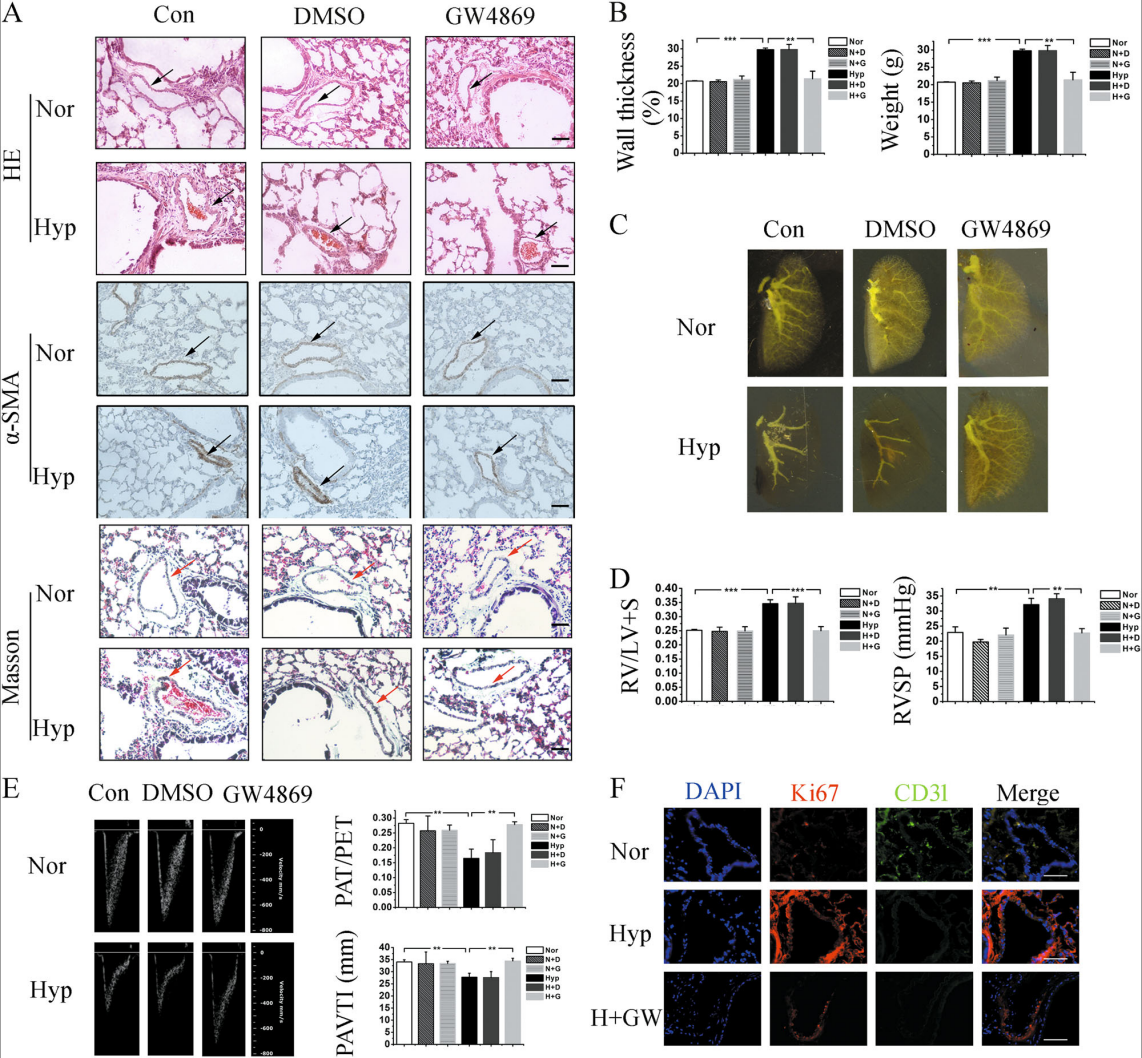
GW4869 reversed the progression of PH induced by hypoxia. **a** The morphology of pulmonary arterial was examined by HE staining, expression of α-SMA, and masson stain. Black and red arrows indicate pulmonary artery. Scale bar = 100 μm. **b** GW4869 administration prevented the increase in wall thickness caused by hypoxia, **c** decreased the pulmonary vascular density, **d** reducesd the ratio of RV weight (RV/LV+S), and mean RVSP, and **e** increased the PAT/PET ratio and PAVTI. *n* = 6. **f** Immunofluorescence staining of Ki67 demonstrated that GW4869 inhibited the overproliferation induced by hypoxia. Scale bar = 50 μm. Con, control; Hyp, hypoxia; Nor, normoxia; N+D, normoxia plus DMSO; H+D, hypoxia plus DMSO; N+G, normoxia plus GW4869; H+G, hypoxia plus GW4869; **p *<* 0.01 and ***p *<* 0.001. All values are presented as mean ± SD

To further verify the role of exosomes in PH, a mouse model of chronic PH was constructed, and the mice were intraperitoneally injected with the exosomes release inhibitor GW4869 or the tail vein was injected with exosomes for 3 weeks as described^18,19^. Vascular remodeling, RVSP, and right ventricular hypertrophy were used to verify the effect of GW4869 on PH. The results indicated that GW4869 was prevented and reversed the vascular remodeling (Fig. 2a, b), reduced the density of pulmonary vasculature (Fig. 2c), elevated right ventricular hypertrophy (the ratio of RV weight to LV weight plus that of the septum: RV/LV+S), mean RVSP (Fig. 2d), decreased the ratio of pulmonary artery acceleration time/pulmonary artery ejection time (PAT/PET), and lowered PAVTI, as compared with the vehicle group (Fig. 2e), while hypoxic PAECs-derived exosomes injection promoted the vascular remodeling, mean RVSP, and right ventricular hypertrophy which induced by hypoxia (Fig. S2). Moreover, immunofluorescence staining of Ki67 revealed that GW4869 inhibited the proliferation of pulmonary arterial vascular (Fig. 2f). Overall, these results showed that exosomes can efficiently modulate the progression of hypoxia-induced PH in vivo.

### Exosomes play a crucial role in PAECs proliferation and migration, which is achieved by influencing the cell cycle distribution

**Fig. 3:**
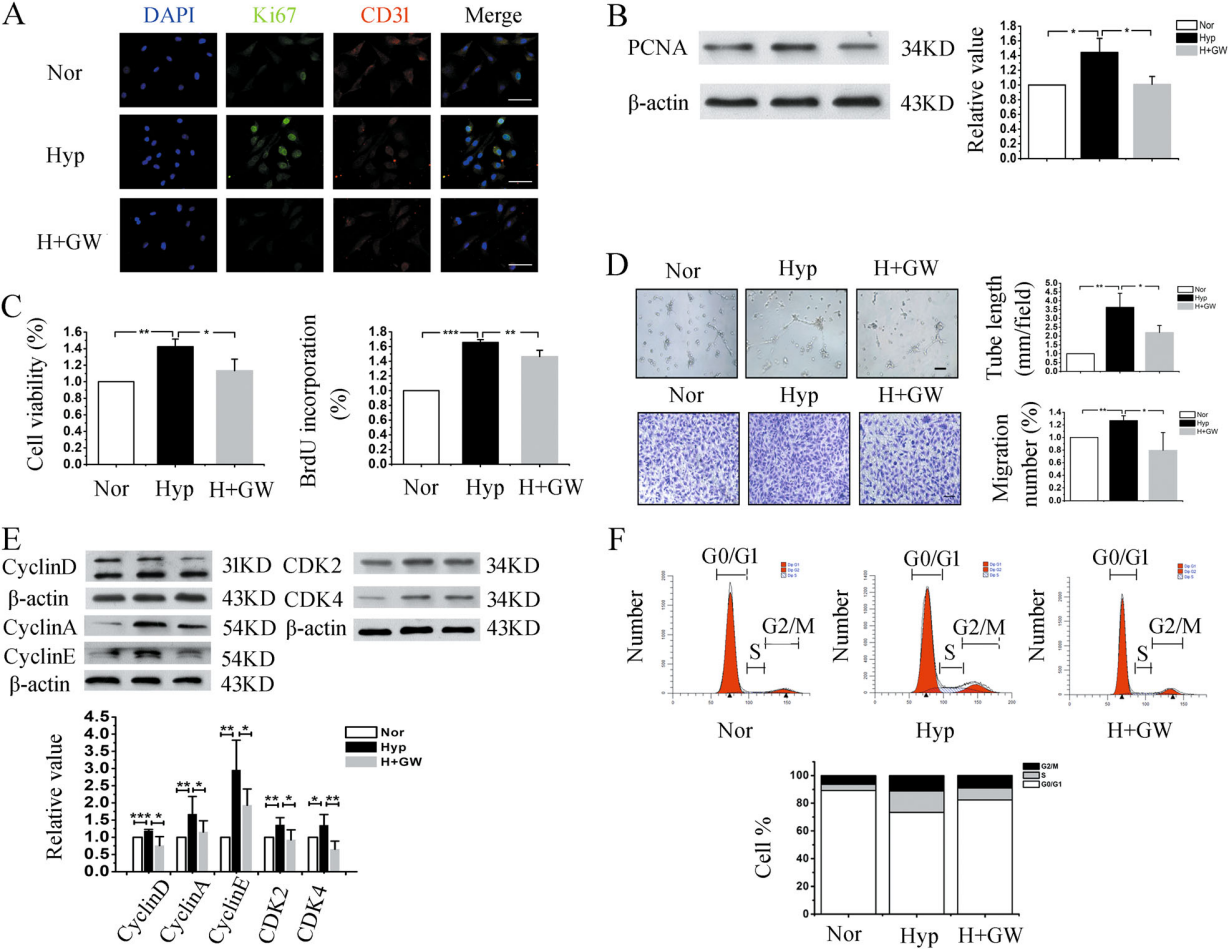
Hypoxia-induced PAECs proliferation and migration were inhibited by GW4869 in vitro. **a** Immunofluorescence staining of Ki67 indicated that GW4869 repressed the hypoxia-induced PAECs growth. Scale bar = 50 μm. **b** The protein expression of PCNA was determined by western blot analysis. **c** Proliferation of PAECs was evaluated by the MTT and BrdU incorporation assays. **d** The tube formation and migration of PAECs were examined after GW4869 treatment. Scale bar = 100 μm. **e**, **f** The number of cells in each phase of the cell cycle was examined by flow cytometry, the protein expression levels of CyclinA, CyclinE, CyclinD, CDK2, and CDK4 were tested under normoxia or hypoxia. Hyp, Hypoxia; Nor, normoxia; H+GW, hypoxia plus GW4869; *p < 0.05, **p < 0.01, and ***p < 0.001. All values are presented as mean ± SD

To investigate the effect of exosomes in vitro under hypoxia, PAECs were treated with GW4869 at a concentration of 10 μM to inhibit exosomes release^20^. As shown in Fig. 3a–c, GW4869 administration restrained the proliferation of PAECs. Moreover, GW4869 reversed the increase in tube length and migration of PAECs induced by hypoxia (Fig. 3d), while there is no significant difference between normoxia and GW4869 pretreatment (Fig. S3). We further investigated whether exosomes promoted angiogenesis via affecting the cell cycle distribution by western blot and flow cytometry analyses, as shown in Fig. 3e, f. GW4869 inhibited the increased number of PAECs entering the S and G2/M phases and arrested the PAECs in the G0/G1 phase. Also, the cell cycle marker proteins induced by hypoxia were suppressed. These results are consistent with the pro-proliferation function of exosomes in vivo.

### 15-LO2/15-HETE expression is upregulated in exosomes under hypoxia, which affected exosomes synthesis and release

**Fig. 4:**
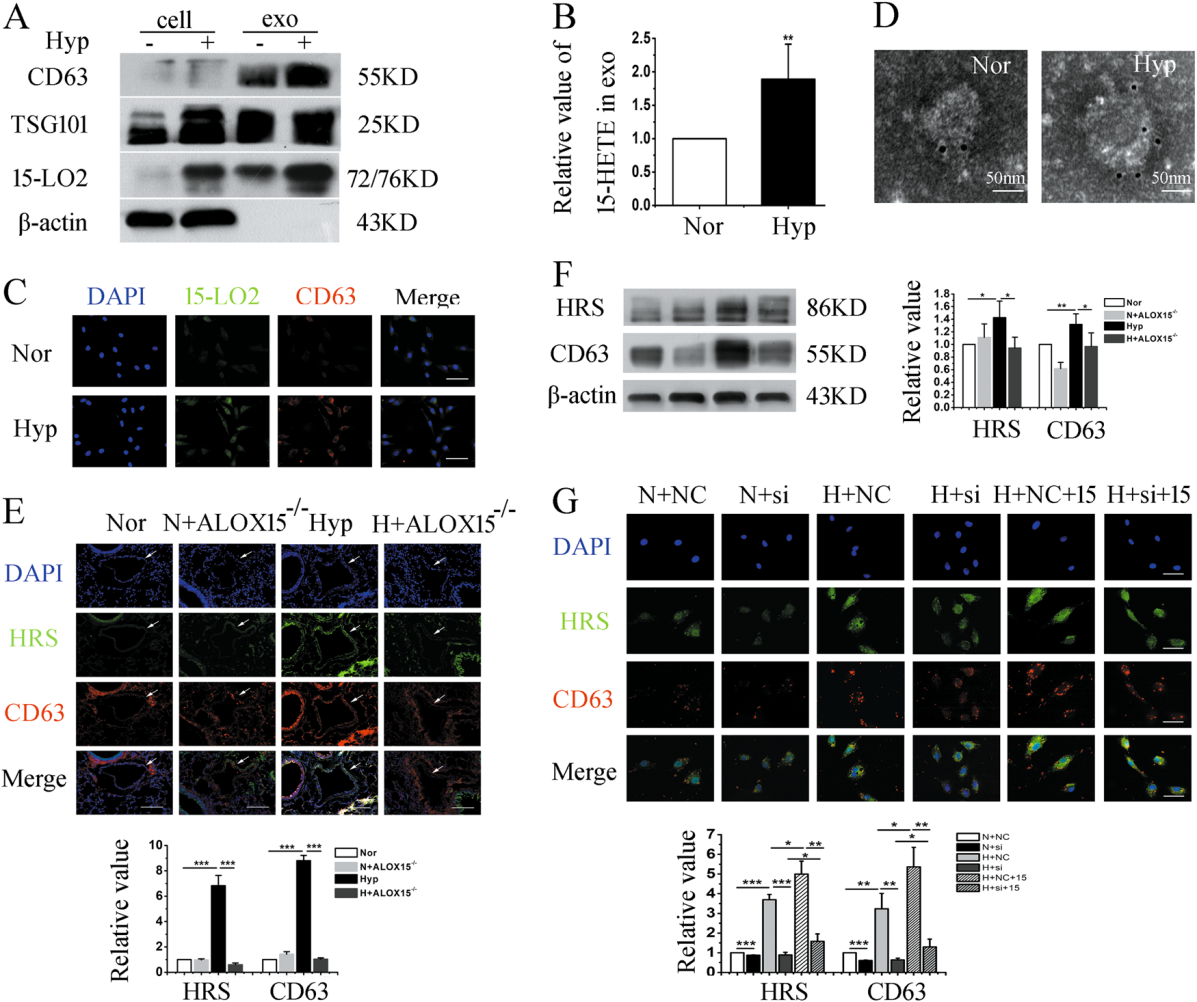
Hypoxia enhanced the expresssion of 15-LO2/15-HETE in exosomes. **a** The expression levels of CD63, TSG101, 15-LO2, and β-actin in the cell lyses and exosomes. **b** The level of 15-LO2 production, 15-HETE was abundantly expressed in exosomes secreted from hypoxic PAECs. **c** PAECs were exposed to normoxia or hypoxia for 24 h and the colocalization between 15-LO2 and CD63 was determined by immunofluorescence. Scale bar = 50 μm. **d** Exosomes extracted from PAECs were prepared for immuno-electron microscope assay. The image showed 15-LO2 was concentrated in the exosomes. Scale bar = 50 nm. **e**, **f** The expression levels of HRS and CD63 in 15-LO knockout or wild-type mice were examined by immunofluorescence and western blot. White arrows indicate pulmonary artery. Scale bar = 100 μm. **g** Immunofluorescence analysis of HRS and CD63 in relative conditioned PAECs. Scale bar = 50 μm. Cell, cell lyses; exo, exosomes; Nor, normoxia; Hyp, hypoxia; N+NC, normoxia plus negative control; N+si, normoxia plus siRNA of 15-LO2; H+NC, hypoxia plus negative control; H+si, hypoxia plus siRNA of 15-LO2; H+NC+15, hypoxia plus negative control and 15-HETE; H+si+15, hypoxia plus siRNA of 15-LO2 and 15-HETE; H + ALOX15^*−/−*^, 15-LO knockout mice under hypoxia; N+ALOX15^*−/−*^, 15-LO knockout mice under normoxia. *p < 0.05, **p < 0.01, and ***p < 0.001. All values are presented as mean ± SD

Given the marked function of exosomes in PH, we analyzed the contents in the exosomes. The 15-LO2 enzyme catalyzes arachidonic acid into 15-HETE^21^, which plays an important role in hypoxia-induced pulmonary vascular remodeling and pulmonary vasoconstriction, with relevance to PH^15,22,23^. As reported in our previous study, hypoxia upregulates 15-LO2 expression in PAECs^21^. However, the relationships between 15-LO2 and exosomes are still unknown. Thus, we evaluated the level of 15-LO2/15-HETE in exosomes secreted by PAECs (Fig. 4a, b). The results showed that 15-LO2/15-HETE was increased in exosomes under hypoxia. Immunofluorescence staining revealed the colocalizations of 15-LO2 and CD63 (a marker of exosomes) in PAECs (Fig. 4c). At the same time, immuno-electron microscope assay exhibited that 15-LO2 was concentrated in the exosomes (Fig. 4d). In addition, to elucidate the interactions between exosomes and 15-LO2, PAECs were pretreated with siRNA against 15-LO2 or 15-HETE, and exosomes synthesis and secretion were determined by immunofluorescence staining (Fig. 4g). Furthermore, exosomes synthesis and secretion were detected in 15-LO knockout mice. As shown in Fig. 4e, f, inhibition of 15-LO2 expression decreased the protein levels of CD63 and HRS, indicating that 15-LO2 affects exosomes synthesis and secretion.

### The STAT3 signaling pathway is mediated by exosomes and 15-LO2

**Fig. 5:**
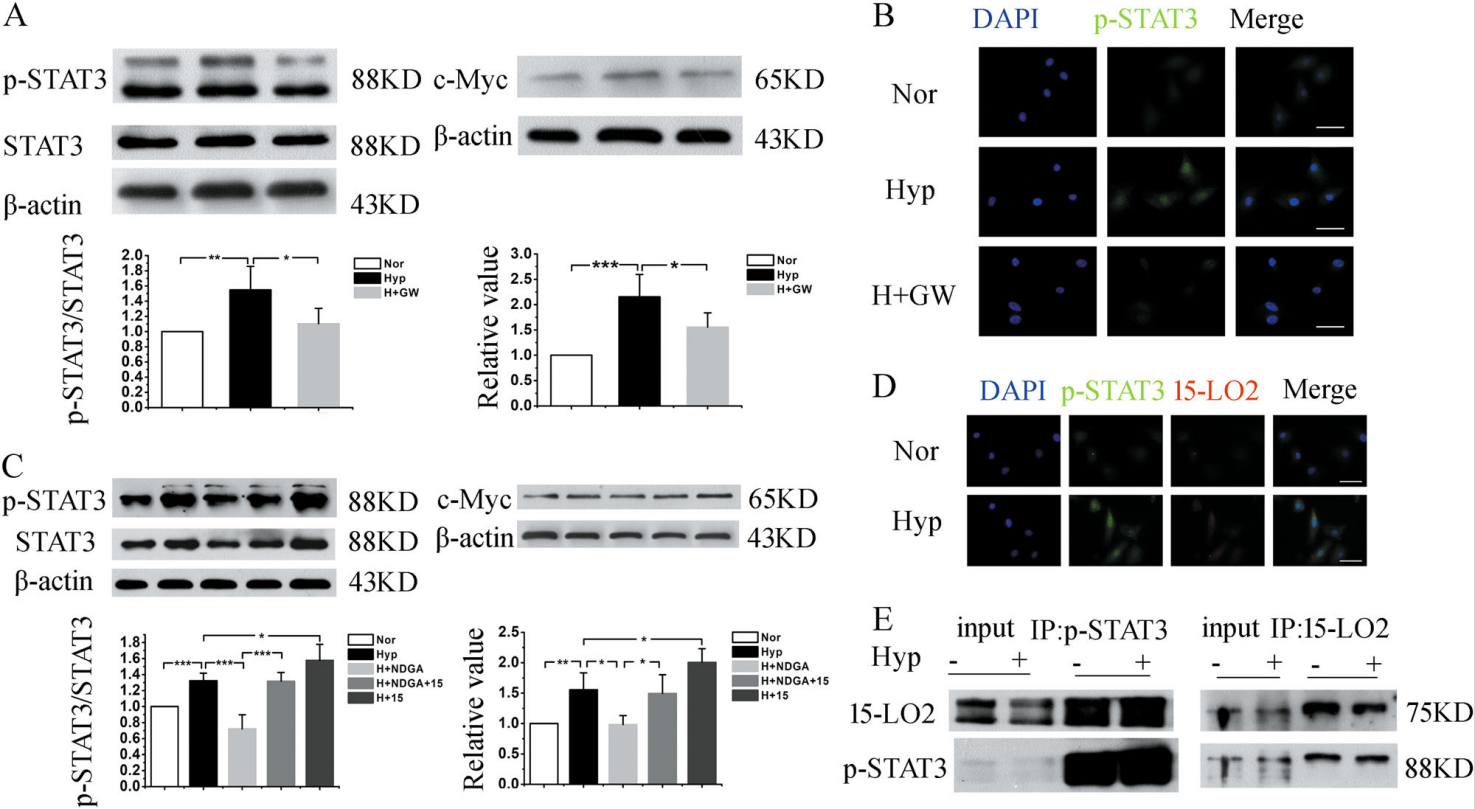
15-LO2-containing exosomes modulated the phosphorylation of STAT3. **a** After treatment with GW4869, the phosphorylation of STAT3 and c-Myc were examined by western blot analysis. **b** Immunofluorescence analysis of p-STAT3 was done after GW4869 administration. **c** PAECs were treated with NDGA or 15-HETE, and the protein levels of p-STAT3 and c-Myc were assessed by western blot analysis. **d** The colocalization of 15-LO2 and p-STAT3 was determined with immunofluorescence assay. **e** PAECs were exposed to normoxia or hypoxia for 24 h and the whole cell lysates were extracted for co-immunoprecipitation with anti-15-LO2 or anti-p-STAT3, followed by probing with anti-p-STAT3 or anti-15-LO2, respectively. Nor, normoxia; Hyp, hypoxia; H+GW, hypoxia plus GW4869; H+NDGA, hypoxia plus NDGA; H+NDGA+15, hypoxia plus NDGA and 15-HETE; H+15, hypoxia plus 15-HETE. Scale bar = 50 μm.*p < 0.05, **p *<* 0.01, and ***p *<* 0.001. All values are presented as mean ± SD

Although, numerous investigators have demonstrated the pivotal role of STAT3 signaling in PH^24,25^, there is no report on the interaction of 15-LO2 or exosomes with STAT3 in PAECs under hypoxia. First, PAECs were pretreated with GW4869. As shown by the western blot and immunofluorescence analyses, hypoxia upregulated the expressions of phosphorylation of STAT3 (p-STAT3), c-Myc, and 15-LO2, while were diminished by GW4869 (Fig. 5a, b and Fig. S5a). Consistently, administration of NDGA (an inhibitor of 15-LO2) abolished the upregulation of p-STAT3 induced by hypoxia (Fig. 5c, d), which had no effect under normoxia (Fig. S5b). Moreover, the co-immunoprecipitation assay demonstrated that there was an interaction between 15-LO2 and STAT3 (Fig. 5e). Overall, these results indicate that 15-LO2-containing exosomes are responsible for the phosphorylation of STAT3 and the expression of the STAT3 target gene c-Myc.

### Proliferation of PAECs induced by the elevation of 15-LO2 is mediated by the STAT3 signaling pathway

To determine whether the STAT3 signaling pathway regulates 15-LO2-containing exosomes-induced angiogenesis, PAECs were treated with 15-HETE and Stattic, an inhibitor of STAT3, and subjected to immunofluorescnce staining for detection of tube formation and migration. The results showed that addition of 15-HETE did not reverse the reduced expression of Ki67 (Fig. S1a), tube formation (Fig. S1b), or Stattic-induced migration of PAECs (Fig. S1c), which suggest that the STAT3 signaling pathway is the downstream of 15-LO2 and involved in the proliferation of PAECs.

### Ubiquitination may be involved in the process that 15-LO2 sorted into exosomes

**Fig. 6:**
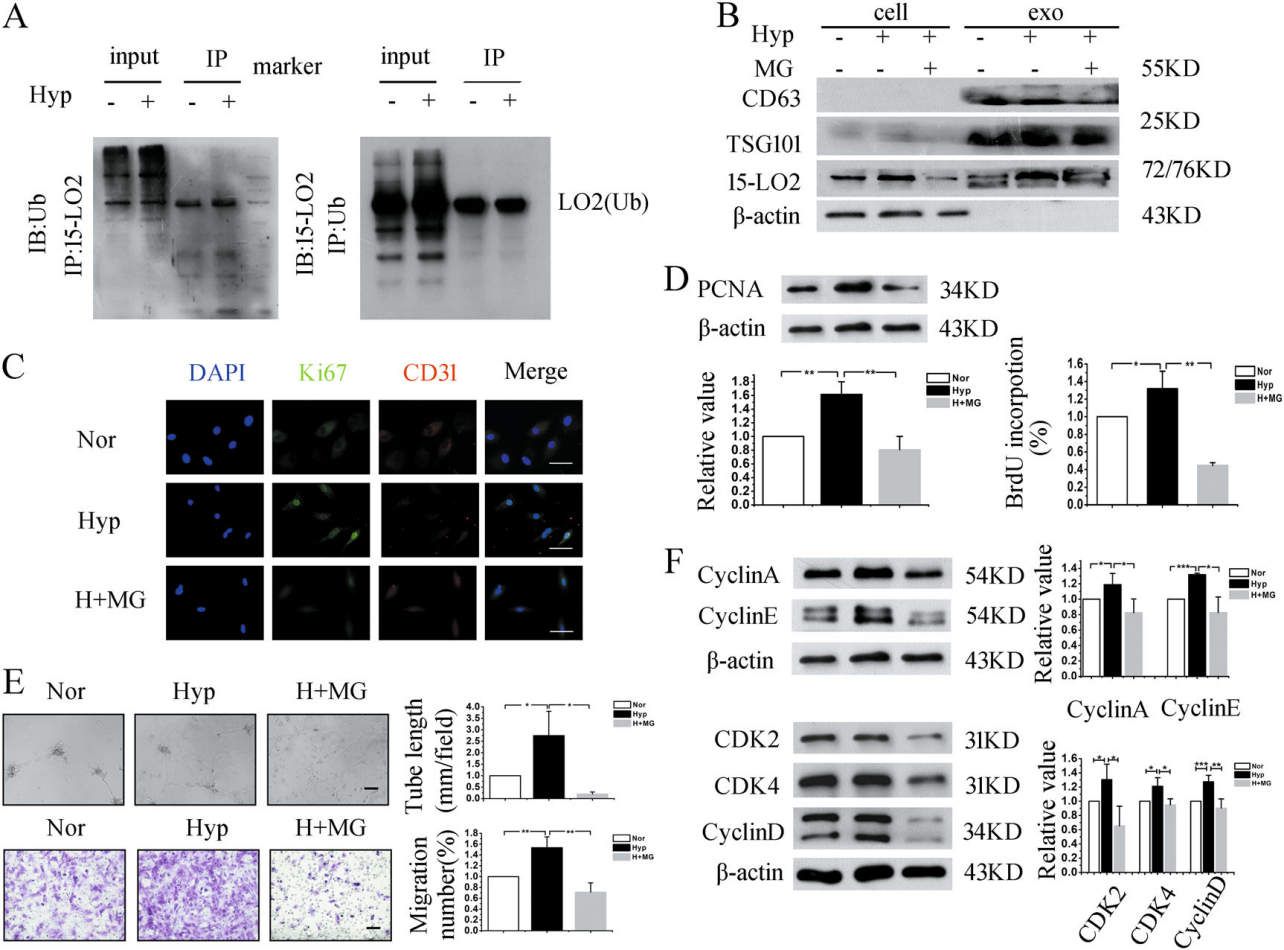
15-LO2 ubiquitination may facilitate its sorting into exosomes in hypoxic PAECs. **a** PAECs were exposed under normoxia or hypoxia for 24 h and then lysed. The cell lysates were immunoprecipitated with anti-ubiquitin antibody or anti-15-LO2 and then blotted with anti-15-LO2 or anti-ubiquitin antibody. **b** PAECs were treated with or without MG-132 (1 μM) for 24 h, and the expression levels of 15-LO2, CD63, TSG101, and β-actin were examined in cell lyses or exosomes. **c** The proliferation of PAECs was examined by immunofluorescence analysis of Ki67 after MG-132 treatment. Scale bar = 50 μm. **d** The proliferation of PAECs was determined by western blot and BrdU incorporation assays. **e** Tube formation and migration of PAECs were inhibited by MG-132, as compared to hypoxia group. Scale bar = 100 μm. **f** The protein expression levels of CyclinA, CyclinD, CyclinE, CDK2, and CDK4 were determined after MG-132 administration. IP, immunoprecipitation; IB, immunoblotting; Ub, ubiquitin; Nor, normoxia; Hyp, hypoxia; H+MG, hypoxia plus MG-132; cell, cell lyses; exo, exosomes. *p < 0.05, **p *<* 0.01, and ***p *<* 0.001. All values are presented as mean ± SD

Exosomes originate in multivesicular bodies (MVBs), and it is thought that an essential post-translational modification which labels protein cargoes to be incorporated into exosomes is ubiquitylated and recognized by the ESCRT apparatus of MVBs^26,27^. We next investigated the mechanisms underlying the enrichment of 15-LO2 in exosomes secreted from hypoxic PAECs. As shown in Fig. 6a, ubiquitinated 15-LO2 was detected in PAECs, while treatment with ubiquitin-protease system inhibitor MG-132 dramatically suppressed cell proliferation (Fig. 6c, d), tube formation, and migration (Fig. 6e). To clarify whether ubiquitinated 15-LO2 promoted angiogenesis by affecting the cell cycle progression, western blot was performed. As shown in Fig. 6f, cell cycle marker proteins were reduced by MG-132. Furthermore, expression of 15-LO2 was detected in the PAECs and exosomes. Strikingly, the 15-LO2 protein was inhibited by MG-132 both in PAECs and exosomes (Fig. 6b). Taken together, these results demonstrate that 15-LO2 sorting into exosomes may be regulated by its ubiquitination.

### The deletion of 15-LO2 reverses the process of PH in vivo

To further verify the role of exosomal 15-LO2 in PH in vivo, we constructed a transgenic mouse expressing dominant-negative 15-LO (ALOX15^−/−^). When the mutation was activated for 4–6 weeks after birth, the mice were then placed into a chronic hypoxic environment. After 21 days, vascular remodeling, RVSP and right ventricular hypertrophy were performed to determine the effect of 15-LO2 in PH. As expected, the deficiency of 15-LO significantly prevented vascular remodeling, right ventricular hypertrophy (RV/LV+S), elevation of RVSP, low density of pulmonary vasculature, reduced data of PAVTI, and decreased PAT/PET ratio induced by hypoxia (Fig. S4a–e).

## Discussion

The results of the present study showed that hypoxia increased the secretion of exosomes in PAECs, which is involved in the progression of hypoxia-induced PH. 15-LO2 was highly expressed and enriched in exosomes secreted by hypoxic PAECs, leading to a robust increase in their neighboring recipient PAECs or themselves’ proliferating in vitro by activating the STAT3 signaling pathway. Moreover, PAECs proliferation was alleviated by inhibiting 15-LO2 ubiquitination under hypoxia. These findings shed new insight into the effect of PAEC secretion of 15-LO2-containing exosomes on recipient PAECs proliferation in a paracrine or autocrine manner, which may predict the etiology of PH.

Exosomes are a series of small vesicles that regulate various physiological and pathological processes, such as immune response, signal transduction, invasion, and metastasis of tumor development^4,28–32^. However, the role of exosomes in hypoxia-induced PH was not fully explored. The present study administrated that hypoxia promoted exosomes secretion in PAECs, and GW4869, the exosomes release inhibitor, restrained the hypoxia-induced proliferation and migration of PAECs, indicating that hypoxia-induced overproliferation and subsequent vascular remodeling are mediated by exosomes. This study also identified that exosomes secreted by anoxic PAECs promoted angiogenesis via affecting cell cycle distribution. In addition, GW4869 was capable of preventing and reversing vascular remodeling, right ventricular hypertrophy (RV/LV+Septum), and reduced density of pulmonary vasculature induced by hypoxia. However, hypoxic PAECs-derived exosomes could promote these parameters in vivo. Overall, our data suggested that exosomes secreted by hypoxic PAECs can efficiently modulate the progression of hypoxia-induced PH. Therefore, our results proved solid evidence that hypoxia-induced the secretion of exosomes in PAECs play an important role in the progression of hypoxia-induced PH.

It has been documented that extracellular vesicles from mice with monocrotaline-induced pulmonary hypertension (PH) induce PH in healthy mice, and the exosomes from mesenchymal stem cells (MSCs) hinder the development of hypoxic PH^11,33^. However, the cell origin of PAECs and functional mechanism of exosomes in PH have not been investigated. The results of this study further clarified an essential role of exosomes in hypoxia-induced PH in vivo and in vitro. In contrast to previous studies^11,33^, we found that hypoxia promoted the secretion of a series of vesicles, which were identified by electromicroscopy as exosomes with a median diameter of ~140 nm and measurement of the exosomal markers TSG101, and CD63^34,35^. Moreover, the exosomes in PAECs exerted a hyperproliferative effect in a paracrine or autocrine manner. Therefore, our results reveal a new cell origin of functional exosomes, which further explore the findings of previous reports. To our best knowledge, this is the first systematic study to confirm the role of exosomes secreted from PAECs on the progression of hypoxia-induced PH.

Emerging evidences demonstrated that exosomes contained numerous lipids, proteins and miRNAs, and function by directly delivering these functional contents to the recipient cells^36,37^. Recently, Ferrer et al. have indicated the key role of exosomal protein TCTP in pulmonary arterial hypertension^38^. In addition, our previous studies have shown that the elevated expression of 15-LO2/15-HETE induced by hypoxia played an important role in PH^13–16^, and the cytoplasm distribution of 15-LO2 in PAECs emerged as a crucial candidate of interest. Thus, we hypothesized that 15-LO2 was incorporated into exosomes in the cell cytoplasm and then participated in the regulation of pathogenesis and development of PH. Our data indicated that 15-LO2 was abundantly expressed and enriched in exosomes secreted from hypoxic PAECs, resulting in a robust increase in PAECs proliferation. Further, in vivo treatment with the exosomes secretion inhibitor GW4869 prevented dysfunctional and abnormal remodeling of the pulmonary vasculature. Moreover, 15-LO2 was ubiquitinated under hypoxia, and further inhibition of ubiquitin-proteasome system significantly suppressed PAECs proliferation. Taken together, it is rational to assume that 15-LO2-containing exosomes contribute to the process of PH. However, multiple exosomal contents factors may also impact exosomes-mediated effects on PH (e.g., microRNA).

Given the obvious importance of 15-LO2 in PH, we conducted further studies in the relationships between 15-LO2 and exosomes. As expected, our results showed that 15-LO2 protein was localized in exosomes secreted by PAECs. In addition, 15-LO2 knockdown decreased the protein levels of CD63 and HRS no matter in vitro or in vivo. Suggesting that hypoxia-induced exosomes synthesis and secretion may be regulated by 15-LO2/15-HETE, although these findings must be further investigated in subsequent work.

STAT3 serves as a transcription factor, has been linked to the hyperproliferative effect on PAECs^2^. It is thus possible that STAT3 regards as a key mediator in exosomes-modulated PH. Indeed, our data showed that the highly expressed 15-LO2 promoted STAT3 phosphorylation at Y705, and they interacted directly in enhancing proliferation and migration of PAECs. However, it should be noted that our results do not exclude the possibility that other members of the STAT family can also mediate the proliferative effect of 15-LO2-containing exosomes secreted by hypoxic PAECs. As far as we know, this is a new mechanism of 15-LO2-containing exosomes modulating the process of PH, which may provide a potential therapeutic target for the treatment of this disease.

Previous reports indicated that ubiquitination was an important step for the sorting of some cargo proteins into MVBs^39,40^. In this study, we found that 15-LO2 was highly ubiquitinated under hypoxia. In addition, the ubiquitin protease inhibitor MG-132 significantly suppressed the proliferation and migration of PAECs. Interestingly, the level of 15-LO2 was also downregulated by MG-132 in cell lysates and exosomes. A possible cause of this phenomenon may be that degradation of 15-LO2 is not controlled by ubiquitination. And whether 15-LO2 ubiquitination facilitates its sorting into exosomes and the underlying mechanism still needs further investigation. To our knowledge, this is the first case on the mechanism of cargo proteins sorting into exosomes in PH. Hence these findings highlight new possibilities to control the progression of PH.

In conclusion, this study demonstrated that 15-LO2 expression was upregulation in exosomes secreted by PAECs under hypoxia, which subsequently activated the STAT3 signaling pathway, resulting in PAECs proliferation and migration via affecting cell cycle distribution. This novel mechanism of exosomes-regulated PH offers a new therapeutic insight for the treating and control of PH.

## Electronic supplementary material

Supplementary Information
