# Supplementary material for: Exosomal 15-LO2 mediates hypoxia-induced pulmonary artery hypertension in vivo and in vitro
Source: Cell Death Dis. 2018 Oct 3;9(10):1022. doi: 10.1038/s41419-018-1073-0 (PMC6170379; doi:10.1038/s41419-018-1073-0)
Supplement: Supplementary file 1 — Supplementary Information [file 41419_2018_1073_MOESM1_ESM.docx]

**Materials and clinical samples**

Antibodies against CDK2 (1:400; Santa Cruz Biotechnology), CDK4 (1:400; Santa Cruz Biotechnology), TSG101 (1:400; abcam) 15-LO2 (1:500; Santa Cruz Biotechnology), CD63 (1:1000; abcam), p-STAT3 (1:1000; abcam), UBB (1:1000; abcam), CyclinA (1:500; China), CyclinE (1:400; China) , PCNA (1:400; China), CyclinD (1:500; China) were used in this study, along with the following reagents and kits: 15-HETE (Cayman), nordihydroguaiaretic acid (NDGA) (Cayman), the annexin V-FITC kit (BD, Bedford, MA), BrdU proliferation assay Kit (Millipore, Billerica, MA), GW4869 (Santa Cruz Biotechnology, California, USA), MG-132 (Beyotime, Shanghai, China), Stattic (Selleck, USA), ExoQuickTC Exosome Precipitation Solution (SBI, USA).

**Histological and morphometric analyses**

The lung tissues of mice were fixed in 4% paraformaldehyde for 24 h, and then dehydrated, cleared, and embedded in paraffin wax. The paraffin-embedded tissues were sliced into 5 μm-thick sections and stained with hematoxylin and eosin (HE) or Masson's trichrome stain. For immunohistochemistry, the lung tissues were dewaxed and incubated overnight with α-smooth muscle actin (α-SMA) at a dilution of 1:100. Afterward, the tissues were washed three times with PBS and then incubated with secondary antibodies. Following incubation, the tissue sections were stained with 3, 3-diaminobenzidine (DAB) and restrained with hematoxylin. Images of tissues were captured with a fluorescence microscope (Nikon) equipped with a digital camera.

**Measurement of right ventricular systolic pressure (RVSP) and ventricular weight**

RVSP was measured by right heart catheterization. The catheter was inserted into the right ventricle (RV), and RVSP was continuously recorded for 30 min. For ventricular weight measurement, mice hearts were excised and RV free wall was dissected and weighed. The degree of RV hypertrophy was determined as the ratio of RV weight to left ventricular (LV) weight plus that of the septum (RV/LV + S).

**Microfil perfusion**

The lung tissues were rinsed with normal saline, and then fixed with formalin for several minutes. The vasculature of the fixed lung tissues was infused with microfil (MV-122; Flow Tech, Inc. Massachusetts, USA). Alcohol-methyl salicylate clearing was implemented according to the manufacturer’s instructions.

**Echocardiography**

Mice were anesthetized with 4% chloral hydrate. Echocardiography was performed with a Vevo2100 imaging system (Visual Sonics Inc., Toronto, On, Canada) and a 30MHz probe. The pulmonary arterial velocity time integral (PAVTI), pulmonary arterial pre-ejection time and pulmonary arterial ejection time were obtained from stable images.

**In vivo exosomes treatment**

For in vivo treatment, PAECs-derived exosomes (30μg every 7 days for 21 days) were adoptively transferred into recipient mice via tail vein injection. In the control group, PBS was used.

**Cell isolation and culture**

The study was approved by the Ethical Committee of Laboratory Animals at Harbin Medical University. PAECs were collected from calf lungs, which were obtained from a local slaughterhouse. The detailed method used was previously described[^1^](#_ENREF_1). PAECs were cultured in 20% fetal bovine serum (FBS)-DMEM in a 5% CO2 humidified incubator at 37 °C.

**Small interfering RNA (siRNA) design and transfection**

PAECs were transfected with siRNA, which was designed and synthesized by GenePharma (China). Non-targeted control siRNA was used as a negative control (NC). The siRNA sequences were as follows: 15-LO2 (NM153301.2): 5’-GCAAUGAAGAACGCCAAAUTT-3’ NC: 5’-CCUACGCCACCAAUUUCGU-3’. The transfection methods used previously described[^1^](#_ENREF_1).

**Measurement of 15**-**HETE level**

Exosomes were purified from a culture solution of PAECs, and the amount of endogenous 15-HETE was measured with 15(S)-HETE enzyme immunoassay (EIA) Kit (Catalog No. 534721, Cayman). The results were analyzed with Cayman Chemical Company EIA Tools.

**Exosomes purification, characterization**

For the isolation and quantification of released exosomes, PAECs were cultured to 90% confluence in complete medium. Afterwards, the PAECs were washed twice with PBS, and then incubated in DMEM with 5% exosomes-depleted FBS for 24 h. The culture medium was harvested for exosomes isolation according to the manufacturer’s protocol of ExoQuickTC Exosome Precipitation kit. Exosomes were verified by electron microscopy and western blot analysis. The final exosomes pellet was resuspended in PBS. Exosomes were labeled with PKH26 (Sigma Aldrich) according to the manufacturer’s protocol. After PKH26 staining, the exosomes were collected by ExoQuickTC Exosome Precipitation kit (SBI). Finally PKH26-labeled exosomes were resuspended in PBS.

**Transmission electron microscopy (TEM), immuno-electron microscope (IEM) and NanoSight analysis (NTA)**

For TEM and IEM, the prepared exosomes were resuspended in PBS. Counterstaining and photographing of exosomes were performed by the Electron Microscopy Center of Harbin Veterinary Research Institute. For NTA, exosomes extracted from PAECs were dissolved in PBS, and the measurement was completed by Tai Chang HuaJia commercial co. LTD (China)

**Tube formation assay**

PAECs in 96-well plates (Costar, Corning) were covered with growth factor-reduced Matrigel (BD Biosciences) in a total volume of 30 μl, and then cultured. Next, the required reagents were added to the medium of different wells. Tube formation was photographed under a fluorescence microscope (Nikon).

**MTT assay**

PAECs were cultured in a 96-well plates and then treated with the required reagents in DMEM with 5% FBS. Then, the samples were exposed to hypoxia (3% O2) for 24 h. At the end of the incubation at 37 °C, the PAECs were incubated for another 4 h in medium containing 0.5% 3-[4, 5-dimethylthiazol-2-yl]-2, 5-diphenyl-tetrazolium bromide (MTT). The reaction was terminated by the addition of DMSO to the medium. The absorbance at 540 nm was measured using a spectrophotometer.

**Migration assay**

For the modified Boyden chamber migration assay, PAECs were cultured in the upper chamber of a transwell, which was inserted into 24-well plates. The lower chamber was filled with 20% FBS-DMEM. Migration was measured after incubation in 0.4% crystal violet in 10% ethanol. The number of migrated PAECs was measured by counting the number of stained nuclei per high-power field under a microscope (Nikon).

**BrdU incorporation assay**

After pretreatment with the indicated agents in DMEM supplemented with 5% FBS for 24 h, cultured PAECs in 96-well culture plates were incubated with 5-BrdU labeling solution for approximately 2 h, followed by FixDenat for 30 min. Then, removed the FixDenat solution and added to anti-BrdU-POD solution for 90 min. The antibody conjugate was removed by rinsing with wash solution, and the PAECs were placed in substrate solution.

**Immunofluorescnce staining**

PAECs were cultured on glass coverslips. At 24 h after exposure with the indicated agents, the PAECs were fixed with 4% paraformaldehyde, permeabilized with 0.01% Triton X-100, blocked with 3% normal bovine serum, and then incubated with the following appropriate antibodies at 4 °C overnight: CD31 (1:100; Santa Cruz Biotechnology), Ki67 (1:100; abcam), CD63 (1:100; abcam), p-STAT3 (1:100; abcam), and HRS (1:100; China). After washing with PBS, the PAECs were incubated with the appropriate FITC-conjugated secondary antibody, Cy3-conjugated secondary antibody, and DAPI in the dark. Images were captured by confocal laser scanning microscopy.

**Coimmunoprecipitation**

The samples were lysed in a lysis buffer (Tris 50 mM, pH7.4, NaCl 150 mM, Triton X-100 1%, EDTA 1 mM, and PMSF 2 mM) and then incubated with monoclonal antibody (5 μg). Afterward, the cells lysates were added with Protein A/G beads (Santa Cruz Biotechnology) overnight at 4 ºC. Antibody-protein complexes were washed three times with PBS, then the buffer was removed and the pellet was resuspended in protein loading buffer (2×). The eluted samples were then subjected to western blot analysis.

**Western blot analysis**

Total protein was extracted from PAECs, and then subjected to western blot analysis as described in detail elsewhere[^1^](#_ENREF_1). In short, cell lysates were prepared and the proteins separated on SDS-PAGE and transferred to a nitrocellulose membranes, which was incubated with primary antibodies. Then, the protein bands were visualized by chemiluminescence.

**Flow cytometry analysis**

The PAECs were treated with the indicated reagent, harvested, and fixed in 70% ethanol, and then stained according to the Cycle TEST PLUS DNA Reagent Kit protocol. The proportions of PAECs in the different phases of the cell cycle were calculated from each histogram.

**References**

1 Ma, C. *et al.* Key role of 15-lipoxygenase/15-hydroxyeicosatetraenoic acid in pulmonary vascular remodeling and vascular angiogenesis associated with hypoxic pulmonary hypertension. *Hypertension* **58**, 679-688, doi:10.1161/HYPERTENSIONAHA.111.171561 (2011).

**
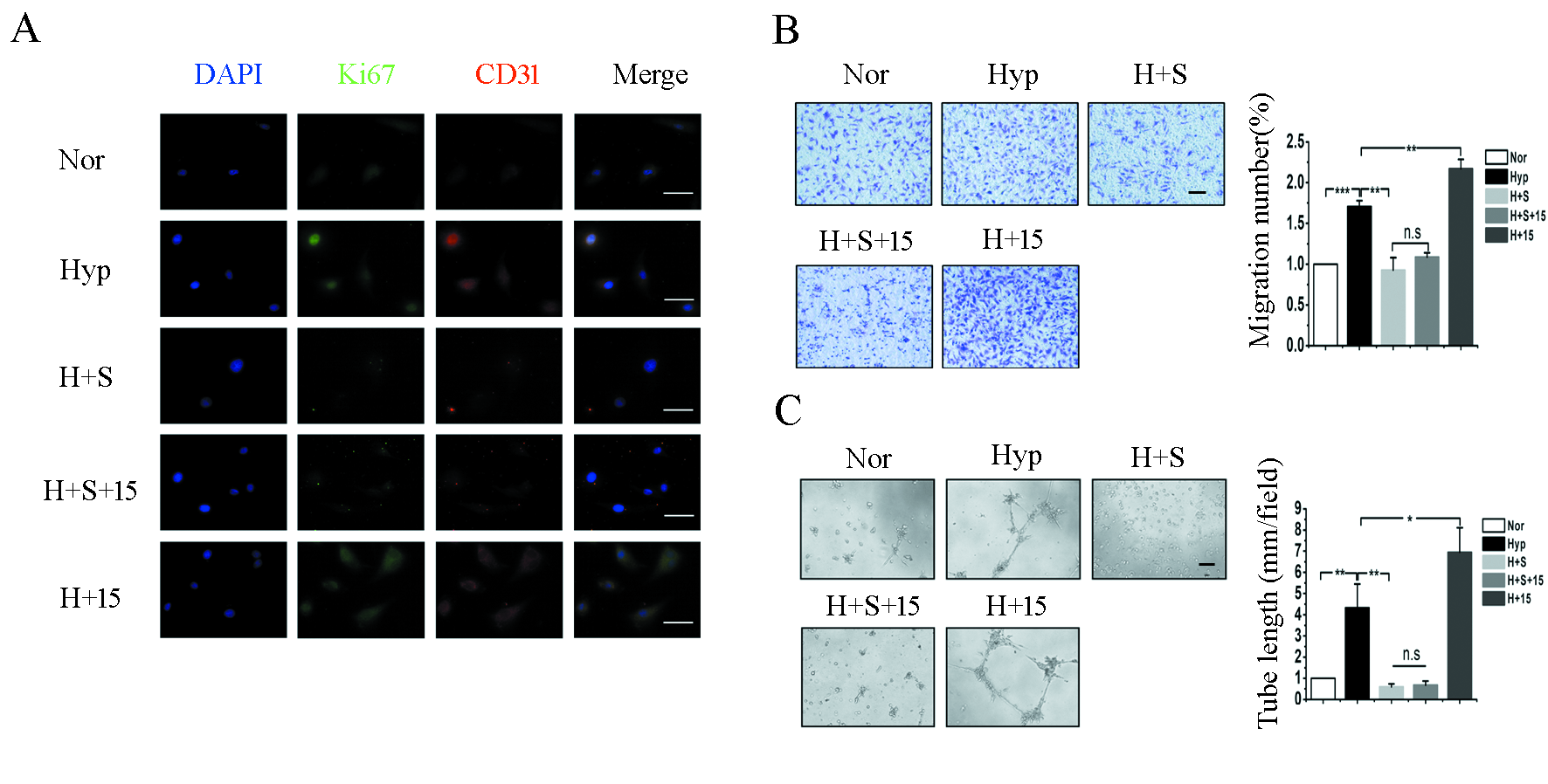
**

**Fig S1. 15**-**LO2 promoted PAECs proliferation and migration via the STAT3 signaling pathway.**

**A,** The proliferation of PAECs was examined by immunofluorescence analysis of Ki67. Scale bars = 50 μm. **B,** The number of migrating PAECs was inhibited by Stattic (5 μM). Scale bars = 100 μm. **C,** Tube formation was repressed by the STAT3 inhibitor Stattic. Scale bars = 100 μm. Nor, normoxia; Hyp, hypoxia; H+S, hypoxia plus stattic; H+S+15, hypoxia plus Stattic and 15-HETE; H+15, hypoxia plus15-HETE. “n.s” means no significance. **p* < 0.05, ***p* < 0.01, and *** *p* < 0.001. All values are presented as mean ± SD.


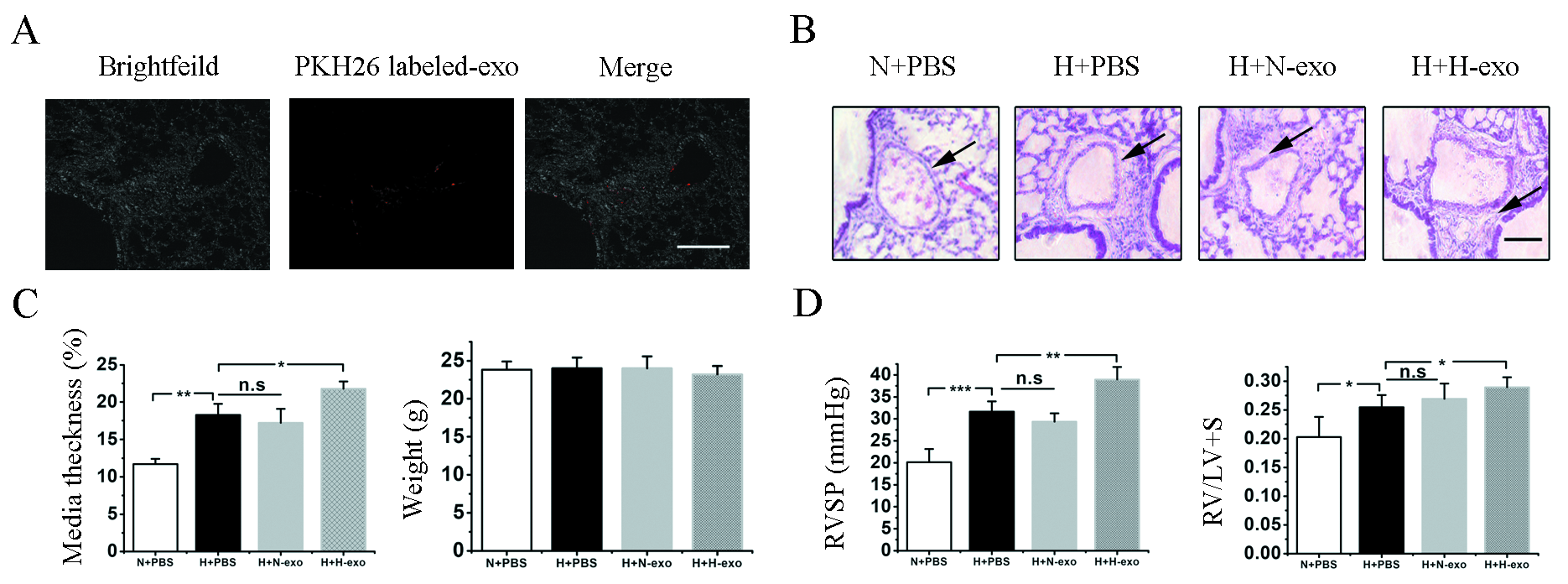


**Fig S2. Exosomes extracted from hypoxic PAECs promoted PH.**

**A,** PKH26 labeled exosomes were displayed in lung tissues of exosomes treated-mice. Scale bar = 100 μm. **B**, The morphology of pulmonary arterial was examined by HE staining. Black arrows indicate pulmonary artery. n = 6. Scale bar = 100 μm. **C, D**, exosomes injection promoted the increase in wall thickness, mean RVSP and the ratio of RV weight (RV/LV+S) caused by hypoxia. N+PBS, normoxia plus PBS; H+PBS, hypoxia plus PBS; H+N-exo, hypoxia plus normoxic PAECs-derived exosomes; H+H-exo, hypoxia plus hypoxic PAECs-derived exosomes. “n.s” means no significance. *p < 0.05, ** *p* < 0.01 and *** *p* < 0.001. All values are presented as mean ± SD.


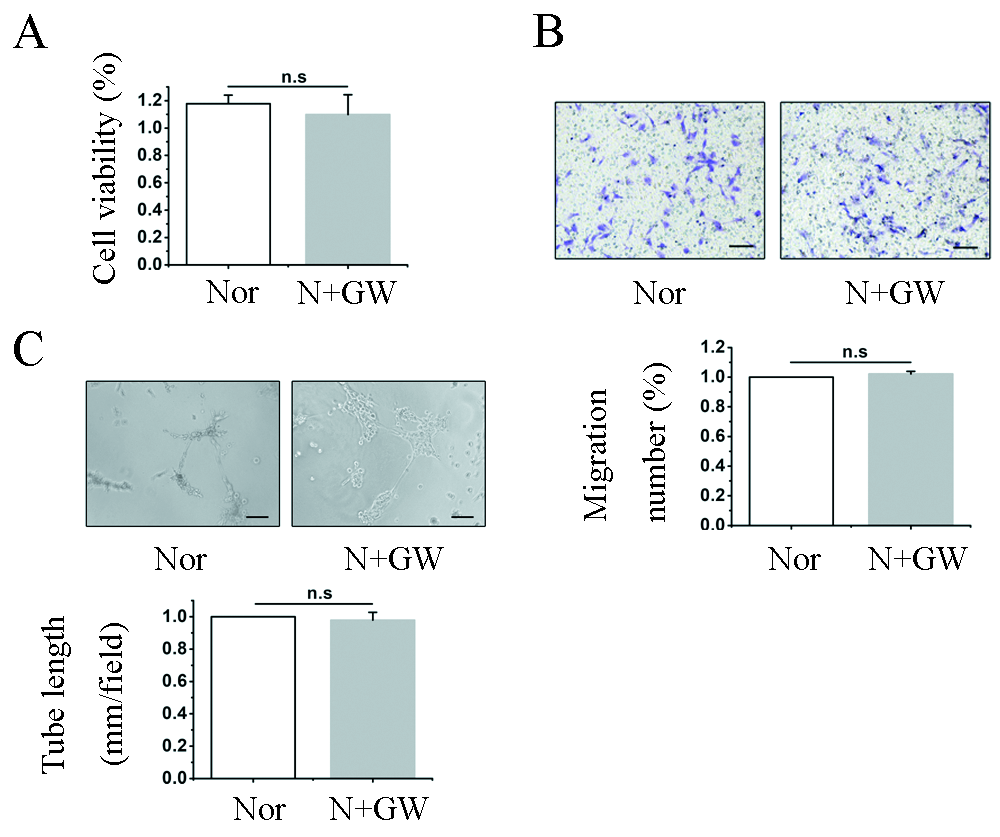


**Fig S3. GW4869 had no effect on PAECs under normoxia.**

**A,** Proliferation of PAECs was evaluated by the MTT assay. **B,** The PAECs migration assay was performed and the number of migratory cells was assessed by crystal violet staining. Scale bar = 100 μm. **C,** The tube formation of PAECs was examined after GW4869 treatment under normoxia. Scale bar = 100 μm. Nor, normoxia; N+GW, normoxia plus GW4869. “n.s” means no significance.


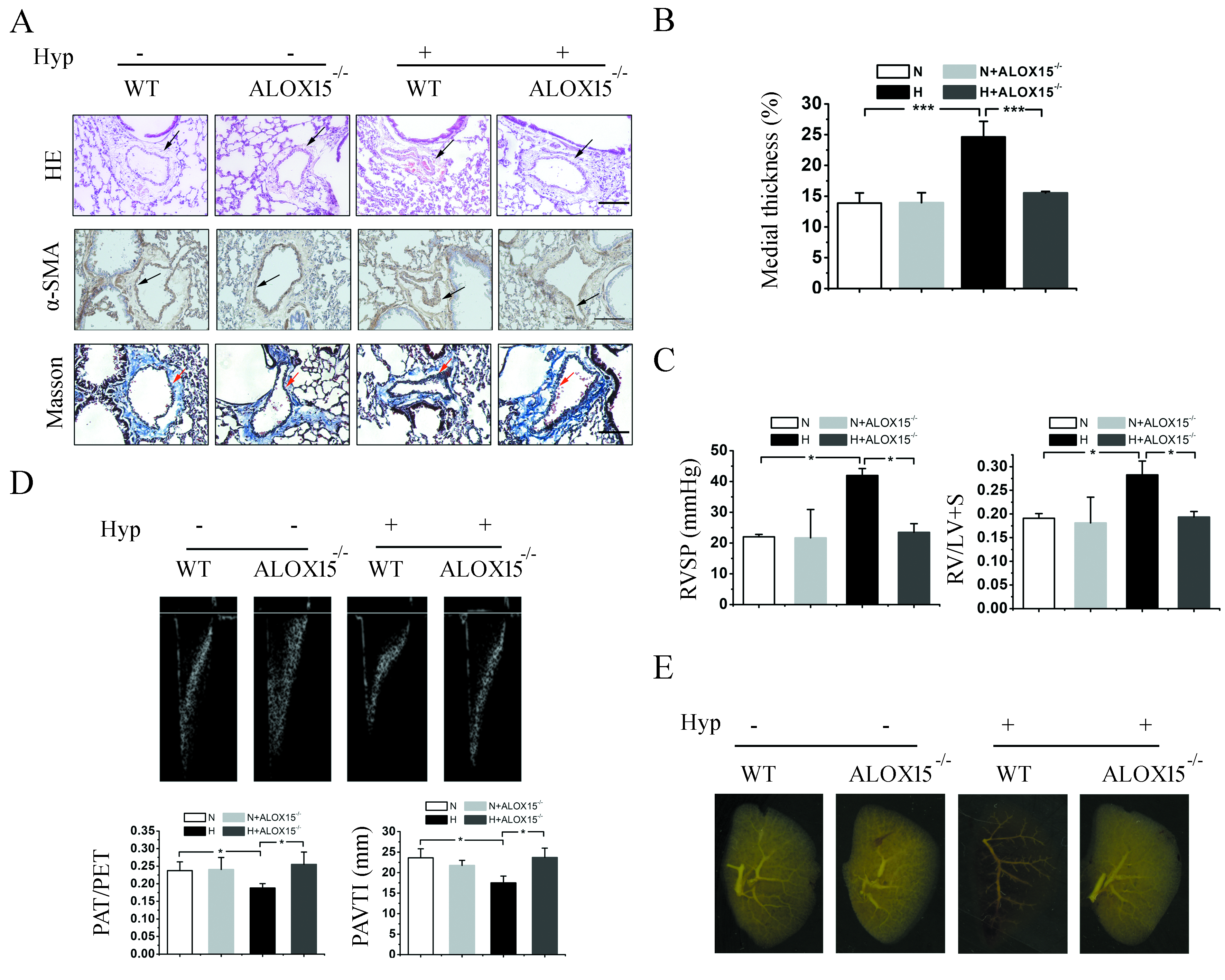


**Fig S4. 15**-**LO knockout reversed hypoxia**-**induced PH.**

**A,** Morphological analysis of pulmonary arterial was examined by HE stain, expression of α-SMA, and masson stain. Black and red arrows indicate pulmonary artery. Scale bars = 100 μm. **B,** 15-LO knockout prevented the increase in wall thickness induced by hypoxia. **C, D** mean RVSP, RV/LV+S, PAT/PET, and PAVTI were measured in 15-LO knockout mice. **E,** 15-LO knockout reversed the low density of pulmonary vasculature induced by hypoxia. N, normoxia; H, hypoxia; WT, wild type; N+ALOX15^-/-^, 15-LO knockout mice under normoxia; H+ALOX15^-/-^, 15-LO knockout mice under hypoxia. **p* < 0.05, ***p* < 0.01 and *** *p* < 0.001. All values are presented as mean ± SD.


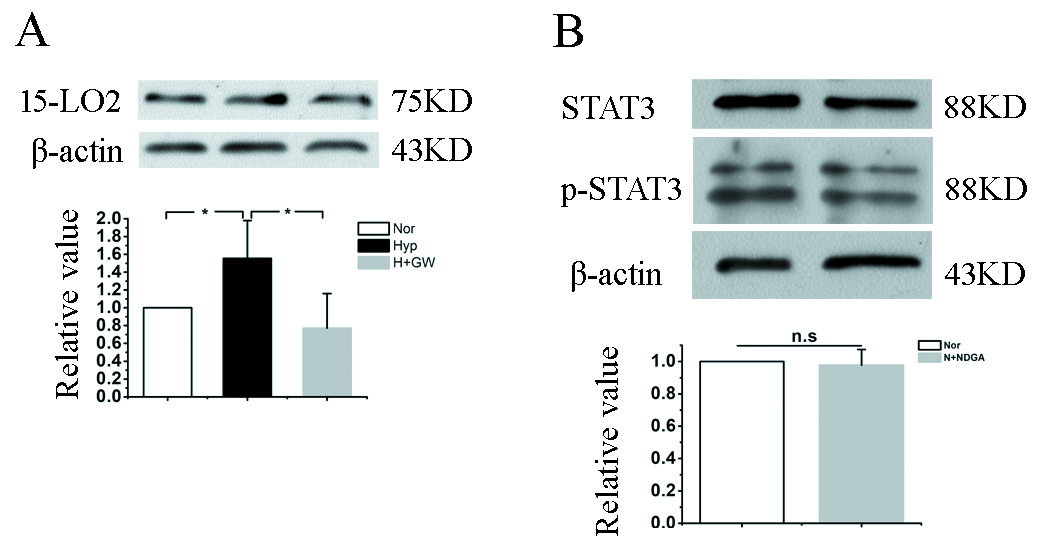


**Fig S5. The effects of GW4869 and NDGA on the relative expression of 15-LO2 and STAT3 signal pathway.**

**A,** The expression of 15-LO2 was detected by western blot. **B,** Western blot of STAT3 and p-STAT3 administrated with NDGA. **p* < 0.05, “n.s” means no significance. All values are presented as mean ± SD.
